# Genome Sequences of 14 Escherichia coli O157:H7 Strains Isolated before and during the Time Frame of the 2018 Multistate Outbreak Associated with Romaine Lettuce

**DOI:** 10.1128/MRA.00458-20

**Published:** 2020-07-16

**Authors:** Eliot Stanton, Sara Wagner, Kelsey R. Florek, Charles W. Kaspar

**Affiliations:** a Department of Bacteriology, University of Wisconsin—Madison, Madison, Wisconsin, USA; b Food Research Institute, Madison, Wisconsin, USA; c Wisconsin State Laboratory of Hygiene, Madison, Wisconsin, USA; University of Maryland School of Medicine

## Abstract

Several outbreaks of Escherichia coli O157:H7 associated with contaminated leafy green vegetables have been documented. Here, we report the draft genome sequences of 14 strains isolated from human patients in the state of Wisconsin during a multistate outbreak in early 2018 that was linked to consumption of romaine lettuce.

## ANNOUNCEMENT

Transmission of enterohemorrhagic Escherichia coli O157:H7 (EHEC) from the bovine reservoir to humans is usually due to fecal contamination of food or water sources (1). EHEC carries an assortment of genes encoding human virulence factors that may include the Shiga-like toxins Stx1 and Stx2 (2). Processing improvements and regulations have led to a decline in outbreaks linked to ground beef (1). Leafy green vegetables typically consumed raw have become an increasingly prominent vehicle of infection. Consumption of romaine lettuce contaminated with EHEC was implicated in a multistate outbreak between March and June 2018 that resulted in 210 reported infections, 96 hospitalizations, and 5 deaths (3). In the current study, sequence data were used to produce draft whole-genome assemblies of 14 EHEC strains isolated from patients in the state of Wisconsin.

The presence of three major phylogenetic lineages within EHEC has been previously demonstrated (4–7). Six PCR markers are used in the lineage-specific polymorphism assay (LSPA-6) that discriminates between lineages I, II, and I/II. LSPA-6 typing was performed *in silico* using reference nucleotide sequences and BLASTn to distinguish the allele sizes using the highest scoring result. The subtypes *stx*_2a_ and *stx*_2c_ were similarly determined *in silico* (8). All isolates belonged to lineage I/II. The majority of strains possessed genes encoding two Stx2 subtypes (Stx2a and Stx2c) and lacked genes encoding Stx1, which is also indicative of lineage I/II. One strain possessed only *stx*_2c_, whereas three strains possessed only *stx*_2a_ (Table 1). Interestingly, EHEC strains isolated from a multistate outbreak associated with spinach in 2006 also belonged to lineage I/II (9).

**TABLE 1:** Accession numbers, assembly statistics, LSPA-6 typing, and *stx* genotyping

Strain	Accession no. for:		Read length (bp)	Total no. of reads	Total sequence length (bp)	Genome coverage (%)	No. of contigs	*N*_50_	GC content (%)	LSPA-6 genotype for marker:						*stx* genotype		
	Assembly	Sequence reads								FolD-*sfmA*	Z5935	*yhcG*	*rbsB*	*rtcB*	*arp*-*iclR*	*stx*_1_	*stx*_2a_	*stx*_2c_
PNUSAE007531	SEKH00000000.1	SRR8499574	250 × 2	1,076,262	5,293,970	98	231	59,778	50.05	2	1	1	1	1	1	−	+	+
PNUSAE010131	SEKI00000000.1	SRR8499573	250 × 2	856,290	5,380,421	78	106	184,501	50.26	2	1	1	1	1	1	−	+	−
PNUSAE010135	SEKJ00000000.1	SRR8499572	250 × 2	868,946	5,358,079	79	104	188,562	50.19	2	1	1	1	1	1	−	+	+
PNUSAE010136	SEKK00000000.1	SRR8499571	250 × 2	711,319	5,434,520	65	117	184,502	50.20	2	1	1	1	1	1	−	+	+
PNUSAE011162	SEKL00000000.1	SRR8499570	250 × 2	1,057,324	5,301,793	96	93	188,562	50.23	2	1	1	1	1	1	−	−	+
PNUSAE011796	SEKM00000000.1	SRR8499569	250 × 2	1,225,680	5,379,475	111	108	188,562	50.23	2	1	1	1	1	1	−	+	+
PNUSAE011802	SEKN00000000.1	SRR8499568	250 × 2	1,164,795	5,377,897	106	103	205,753	50.23	2	1	1	1	1	1	−	+	+
PNUSAE011803	SEKO00000000.1	SRR8499567	250 × 2	905,669	5,384,870	82	104	188,562	50.24	2	1	1	1	1	1	−	+	+
PNUSAE011805	SEKP00000000.1	SRR8499566	250 × 2	712,977	5,386,954	65	108	184,501	50.25	2	1	1	1	1	1	−	+	−
PNUSAE012259	SEKQ00000000.1	SRR8499565	250 × 2	1,079,803	5,373,223	98	95	190,279	50.23	2	1	1	1	1	1	−	+	−
PNUSAE012740	SEKR00000000.1	SRR8499562	250 × 2	1,917,240	5,308,743	174	111	187,686	50.24	2	1	1	1	1	1	−	+	+
PNUSAE013643	SEKS00000000.1	SRR8499561	250 × 2	1,272,417	5,389,036	116	111	184,501	50.25	2	1	1	1	1	1	−	+	+
PNUSAE013919	SEKT00000000.1	SRR8499564	250 × 2	1,008,668	5,254,297	92	125	119,444	50.22	2	1	1	1	1	1	−	+	+
PNUSAE014032	SEKU00000000.1	SRR8499563	250 × 2	672,469	5,293,062	61	97	188,562	50.28	2	1	1	1	1	1	−	+	+

Clinical isolates were grown on blood agar plate (BAP) medium and Sorbitol-MacConkey agar (SMAC) medium and subsequently checked with Difco and SSI O157 antisera. Biochemical assays consisted of triple sugar iron, motility, and the API 20E fast identification system. The presence/absence of the genes *stx*_1_, *stx*_2_, *eae*, and *ehxA* was determined using PCR amplification. The isolates were stored frozen and briefly thawed; approximately 10 μl was then streaked onto Columbia blood agar (Remel). The plates were incubated overnight at 37°C. Growth from a single colony was streaked onto a new plate and incubated overnight. The growth was transferred to 200 μl of UltraPure molecular-grade (Sigma) water containing 180 μl of lysis buffer from a MagNA Pure LC DNA isolation kit III (Roche). The samples were vortexed until homogenous, proteinase K was added (20 μl), and the samples were vortexed again (5 to 10 s). The samples were then heated at 65°C for 10 min. Lysates were transferred into the appropriate wells of a MagNA Pure LC sample cartridge and run according to the manufacturer’s specifications. DNA from the final eluate was quantified by using a Qubit 2.0 fluorometer (Thermo Fisher Scientific) and a NanoDrop 2000 spectrophotometer (Thermo Fisher Scientific). Total genomic DNA (gDNA) from each isolate was used to construct a paired-end sequencing library using the Nextera XT DNA library preparation kit (Illumina). Individual libraries were multiplexed and sequenced on the MiSeq platform (Illumina) using the MiSeq reagent kit v2500 cycle (Illumina). Genome assemblies for each strain were produced using SPAdes v3.11.1 in careful mode and utilizing BayesHammer to perform error correction (10). The draft-genome assemblies were subsequently iteratively corrected using Bowtie v1.2.2 to align the paired-end reads with a maximum insert size of 1,000 bp and Pilon v1.22 (11, 12). Contigs less than 1,000 bp long or possessing k-mer coverage less than 20 were excluded from the final assembly. The genomes were annotated in NCBI PGAP v4.7 (13, 14).

### Data availability.

The sequence data were deposited in GenBank/EMBL/DDBJ under the accession numbers listed in Table 1 and collected under BioProject accession number PRJNA517910.
